# *AHRR* methylation in heavy smokers: associations with smoking, lung cancer risk, and lung cancer mortality

**DOI:** 10.1186/s12885-020-07407-x

**Published:** 2020-09-22

**Authors:** Laurie Grieshober, Stefan Graw, Matt J. Barnett, Mark D. Thornquist, Gary E. Goodman, Chu Chen, Devin C. Koestler, Carmen J. Marsit, Jennifer A. Doherty

**Affiliations:** 1 Department of Population Health Sciences, Huntsman Cancer Institute, University of Utah, 2000 Circle of Hope Drive, Room 4746, Salt Lake City, UT 84112 USA; 2 Department of Biostatistics & Data Science, University of Kansas Medical Center, Kansas City, KS USA; 3 Department of Environmental Health, Rollins School of Public Health, Emory University, Atlanta, GA USA; 4 Program in Epidemiology, Division of Public Health Sciences, Fred Hutchinson Cancer Research Center, Seattle, WA USA; 5 Department of Epidemiology, School of Public Health, University of Washington, Seattle, WA USA; 6 Department of Otolaryngology: Head and Neck Surgery, School of Medicine, University of Washington, Seattle, WA USA

**Keywords:** Lung cancer, Epidemiology, Biomarkers/serum biomarkers, Methylation, *AHRR*, CARET, Mortality

## Abstract

**Background:**

A low level of methylation at cg05575921 in the aryl-hydrocarbon receptor repressor (*AHRR*) gene is robustly associated with smoking, and some studies have observed associations between cg05575921 methylation and increased lung cancer risk and mortality. To prospectively examine whether decreased methylation at cg05575921 may identify high risk subpopulations for lung cancer screening among heavy smokers, and mortality in cases, we evaluated associations between cg05575921 methylation and lung cancer risk and mortality, by histotype, in heavy smokers.

**Methods:**

The β-Carotene and Retinol Efficacy Trial (CARET) included enrollees ages 45–69 with ≥ 20 pack-year smoking histories and/or occupational asbestos exposure. A subset of CARET participants had cg05575921 methylation available from HumanMethylationEPIC assays of blood collected on average 4.3 years prior to lung cancer diagnosis in cases. Cg05575921 methylation β-values were treated continuously for a 10% methylation decrease and as quintiles, where quintile 1 (Q1, referent) represents high methylation and Q5, low methylation. We used conditional logistic regression models to examine lung cancer risk overall and by histotype in a nested case-control study including 316 lung cancer cases (diagnosed through 2005) and 316 lung cancer-free controls matched on age (±5 years), sex, race/ethnicity, enrollment year, current/former smoking, asbestos exposure, and follow-up time. Mortality analyses included 372 lung cancer cases diagnosed between 1985 and 2013 with available methylation data. We used Cox proportional hazards models to examine mortality overall and by histotype.

**Results:**

Decreased cg05575921 methylation was strongly associated with smoking, even in our population of heavy smokers. We did not observe associations between decreased pre-diagnosis cg05575921 methylation and increased lung cancer risk, overall or by histotype. We observed linear increasing trends for lung cancer-specific mortality across decreasing cg05575921 methylation quintiles for adenocarcinoma and small cell carcinoma (*P*-trends = 0.01 and 0.04, respectively).

**Conclusions:**

In our study of heavy smokers, decreased cg05575921 methylation was strongly associated with smoking but not increased lung cancer risk. The observed association between cg05575921 methylation and increased mortality in adenocarcinoma and small cell histotypes requires further examination. Our results do not support using decreased cg05575921 methylation as a biomarker for lung cancer screening risk stratification.

## Background

Exposure to cigarette smoke is associated with altered DNA methylation at thousands of individual cytosine-guanine dinucleotide (CpG) sites across the genome in both blood and lung tissue based on results from at least 73 epigenome-wide association studies (EWAS) [1]. The most consistent association for any CpG with smoking is decreased methylation at cg05575921 in the aryl hydrocarbon receptor repressor gene (*AHRR*), which has been associated with cigarette smoking in whole blood samples in at least 30 EWAS [1]. The cg05575921 locus typically shows the largest absolute difference in methylation by cigarette smoking relative to other individual CpGs [2–11]. Longitudinal studies have shown that decreased methylation of cg05575921 persists in former smokers compared to never smokers, and that methylation gradually increases with time since cessation [5, 11, 12].

Cg05575921 is located in an *AHRR* gene enhancer, and decreased methylation in this region results in increased *AHRR* gene expression in both blood [13, 14] and lung tissue [15–17]. Greater *AHRR* expression inhibits the aryl-hydrocarbon receptor, which among other functions, regulates toxicity of polycyclic aromatic hydrocarbons (PAHs) [18]. Since cigarette smoke contains PAHs, it has been hypothesized that decreased *AHRR* methylation induced by cigarette smoking may be a mediator in lung cancer development [19]. Several epidemiologic studies support this hypothesis and report that a low level of cg05575921 methylation is associated with increased lung cancer risk [4, 9, 19–22]. However, these reports all include light and never smokers. While decreased cg05575921 methylation has been reported to be associated with all-cause mortality [9, 12], the relationship between pre-diagnosis cg05575921 methylation and mortality in lung cancer cases is less clear. One case-cohort study reported increased lung cancer-specific mortality [23], but results were not presented by histotype, which could limit the examination of associations among tumor subgroups with known differences in treatment response and mortality. To our knowledge, no studies to date have examined associations with pre-diagnosis cg05575921 methylation and mortality, all-cause or lung cancer-specific, among lung cancer cases.

Since a low level of cg05575921 methylation is highly correlated with increased smoking exposure, and has been reported to be associated with lung cancer risk, it is an appealing marker to examine for risk stratification for lung cancer screening. Since 2014, the United States Preventive Services Task Force (USPSTF) has recommended annual lung cancer screening for individuals aged 55–80 years who have at least 30 pack-year smoking histories and are current or former smokers who quit within the past 15 years [24]. An updated 2020 draft USPSTF recommendation statement broadens screening eligibility to include those aged 50–80 with 20 or more pack-year smoking histories, still among current or former smokers who quit within the past 15 years [25]. In order for a biomarker to improve lung cancer screening risk stratification by minimizing false-positive screens, it must be associated with lung cancer risk among individuals who are eligible for screening. We sought to disentangle the relationships between cg05575921 methylation, lung cancer risk, and lung cancer mortality in a nested case-control study of heavy smokers generally representative of a lung cancer screening-eligible population.

## Methods

Our study includes a subset of participants from the multicenter β-Carotene and Retinol Efficacy Trial (CARET) [26]. CARET was a randomized, double-blinded, placebo-controlled trial designed to assess the safety and efficacy of daily β-carotene and retinyl palmitate supplementation in heavy smokers at high risk of developing lung cancer [26–28]. From 1985 to 1994, CARET enrolled 14,254 men and women ages 50–69 years who were current or former smokers (quit ≤ 6 years prior to enrollment) with ≥ 20 pack-year cigarette smoking histories. Men with occupational asbestos exposure ages 45–69 years who were current or former smokers (quit ≤ 15 years prior to enrollment) were also enrolled (*n* = 4060). Smoking status, smoking history, and other risk factors were collected via annual questionnaires. Whole blood samples were collected at visits between 1994 and 1997. The intervention was stopped in 1996 due to higher lung cancer incidence and overall mortality rates in the intervention versus placebo arm.

Within our larger matched case-control study designed to examine genetic factors and lung cancer risk described in [29], we generated whole-genome DNA methylation data for 350 lung cancer cases identified during active follow-up between 1985 and 2005, and one matched control per case. The case-control pairs were matched on enrollment characteristics including age (±4 years) and smoking status, as well as sex, race/ethnicity, enrollment year (±2 years), and history of occupational asbestos exposure. Controls were cancer-free at least as long as their corresponding case through 2005.

DNA was extracted from whole blood using QIAGEN QIAmp DNA Blood Midi Kits (*n* = 348 cases, *n* = 347 controls) and 5PRIME ArchivePure DNA Purification Kits (*n* = 2 cases, *n* = 3 controls). DNA methylation was assayed in a single batch using the Illumina HumanMethylationEPIC BeadArray at the University of Southern California Epigenomics Core Facility following standardized protocols from Illumina, Inc. We performed data quality control, preprocessing, and Noob+β-mixture quantile normalization using the *minfi* and *wateRmelon* Bioconductor packages [30, 31], described in detail previously [32]. Analytical β-values, representing percent methylation, were obtained for the cg05575921 locus.

Since blood was collected at post-enrollment study visits, and DNA methylation is influenced by age and smoking status, we re-matched among the 350 case-control pairs using age (±5 years) and smoking status (current or former) at blood draw, rather than at enrollment, as well as sex, race/ethnicity, enrollment year (±2 years), asbestos exposure, and duration of follow-up. A total of 322 case-control pairs were able to be re-matched, but three pairs missing data on body mass index (BMI) were removed, resulting in 319 pairs in our previous study [32]. For the present analysis, we included the three pairs missing BMI, but we discovered that there were six mismatched pairs that were removed for the present analysis. Analyses examining cg05575921 methylation and risk of lung cancer therefore include 316 matched case-control pairs, with blood collected on average 4.3 years prior to diagnosis for the cases. Mortality analyses were performed for all 350 lung cancer cases diagnosed through 2005, plus 22 controls who developed lung cancer during passive follow-up from 2005 to 2013; blood was collected on average 4.9 years prior to diagnosis for this larger case group.

### Statistical analysis

We categorized cg05575921 percent methylation into quintiles, with quintile 1 (Q1, referent) containing the top 20% of percent methylation values (i.e., hypermethylation), and Q5 containing the lowest 20% of percent methylation values (i.e., hypomethylation). Cut points for cg05575921 quintile methylation for the lung cancer risk analyses are based on the distribution of cg05575921 methylation in the controls. We used ordinal linear regression to assess linear trends of association between cg05575921 methylation quintiles and continuous participant characteristics including age, BMI, cigarettes per day in current smokers, pack years smoked, and years since cessation in former smokers. We assessed linear trends in proportions of strata for discrete participant characteristics, including race, sex, smoking status, and occupational asbestos exposure, as well as stage and histotype (adenocarcinoma, squamous cell carcinoma, or small cell carcinoma) across cg05575921 methylation quintiles using Cochran-Armitage Trend tests, or Fisher’s Exact tests for variables with at least 50% of cells containing expected counts of less than five per cell.

We evaluated associations between continuous decreasing cg05575921 methylation and lung cancer risk using multivariable-adjusted logistic regression models conditioned on matching factors. In addition to a priori selected adjustment for continuous age at blood draw (to reduce residual confounding by age) and methylation-derived estimated blood cell type proportions [33, 34], adjustment variables were assessed for inclusion based on biologic plausibility and/or if their addition to age- and estimated cell type-adjusted conditional logistic regression models for all lung cancer cases resulted in a ≥ 10% change in the estimated odds ratio for either quintile or continuous 10% decreased cg05575921 methylation. Final risk models were adjusted for age at blood draw, estimated blood cell proportions, and cigarettes per day at blood draw. We performed the same analysis restricted to the 242 matched pairs where both the case and control would have been eligible for lung cancer screening based on age (55–80 years) and smoking (≥ 30 pack years; current or quit < 15 years) per the 2014 USPSTF recommendation statement.

For mortality analyses, quintile cg05575921 percent methylation cut points were based on the distribution including all 372 lung cancer cases. We evaluated associations between decreasing pre-diagnosis cg05575921 methylation and lung cancer-specific and all-cause mortality using multivariable-adjusted Cox proportional hazards models with follow-up defined as time between lung cancer diagnosis and death or December 31, 2013, whichever occurred first. We included a strata variable for early, late, or unknown stage to allow for differing baseline hazards since stage at diagnosis is strongly associated with mortality [35]. Continuous age, sex, methylation-derived estimated blood cell type proportions [33, 34], and time between blood draw and diagnosis were a priori selected for adjustment, and additional variables were included based on biologic plausibility and/or if their addition to a priori variable-adjusted Cox proportional hazards models for all lung cancer cases resulted in a ≥ 10% change in the estimated hazard ratio (all-cause or lung cancer-specific) for either quintile or continuous 10% decreased cg05575921 methylation. Final mortality models were adjusted for age at blood draw, sex, estimated blood cell proportions, time between blood draw and diagnosis, smoking status, and years since smoking cessation at blood draw.

We performed a sensitivity analysis excluding the three pairs where either the case or control had DNA extracted by the 5PRIME method. We also examined the possibility of interaction by sex in the mortality models, overall and by histotype, to ensure sound adjustment for sex as a confounder and not an effect modifier in our models. All analyses were performed in SAS 9.4 (Cary, NC). Statistical tests were two-sided and statistical significance testing was performed at a nominal level of *P* < 0.05.

## Results

We observed highly statistically significant linear trends of increasing proportions of current smokers across decreasing cg05575921 methylation quintiles in both lung cancer cases and controls (*P*_case_ = 2 × 10^− 22^, *P*_control_ = 4 × 10^− 25^; Table 1). Striking differences in the proportions of current smokers were observed in quintile five (Q5) compared to Q1 in both cases (90% vs 24%) and controls (89% vs 22%). Similar trends were observed across increasing quintiles with greater total years smoked (*P*_case_ = 0.03, *P*_control_ = 1 × 10^− 8^), fewer years since cessation in former smokers (*P*_case_ = 0.002, *P*_control_ = 0.001), and more cigarettes smoked per day in current smokers (*P*_case_ = 8 × 10^− 5^, *P*_control_ = 0.04). We observed linear associations with increasing quintiles for increasing pack years (only statistically significant among controls: *P*_control_ = 0.004; *P*_case_ = 0.15), decreasing BMI (*P*_case_ = 0.004, *P*_control_ = 0.002), and age at blood draw (only statistically significant among cases: *P*_case_ = 7 × 10^− 5^; *P*_control_ = 0.07). We observed decreasing proportions of individuals with asbestos exposure across increasing quintiles (*P*_case_ = 0.05; *P*_control_ = 0.003). We observed similar linear trends across decreasing cg05575921 methylation quintiles in the full 372 cases examined in the mortality analyses (Additional file 1: Table S1).

**Table 1:** Characteristics of lung cancer cases and controls by quintiles of cg05575921 percent methylation

	All	Q1	Q2	Q3	Q4	Q5	
		(hyper-methylated)				(hypo-methylated)	
Quintile range	34–98	84–98	73–84	64–73	56–64	34–56	
**Lung cancer cases**^a^							
	**(*n*** **= 316)**	**(*n*** **= 59)**	**(*n*** **= 78)**	**(*n* = 59)**	**(*n*** **= 49)**	**(*n*** **= 71)**	***P-*trend**^**b**^
**cg05575921**, % methylation; mean (SD)	69.1 (14.6)	89.6 (3.8)	78.0 (3.1)	68.2 (2.7)	60.0 (2.4)	49.2 (5.0)	–
**Age at blood draw**, years; mean (SD)	64.5 (5.5)	65.9 (5.8)	65.5 (5.5)	64.9 (5.7)	63.8 (4.9)	62.5 (5.3)	7 × 10^−5^
**Current smoker**; No. (%)	205 (65)	14 (24)	38 (49)	44 (75)	45 (92)	64 (90)	2 × 10^−22^
**Years since smoking cessation**^c^; mean (SD)	6.6 (4.8)	8.1 (5.2)	6.4 (4.6)	4.3 (3.9)	5.0 (2.2)	3.7 (2.6)	0.002
**Pack years**; mean (SD)	59.3 (22.5)	55.3 (18.4)	59.0 (25.1)	60.2 (22.3)	60.7 (23.8)	61.3 (22.2)	0.15
**Average cigarettes per day**^d^; mean (SD)	23.3 (12.4)	17.6 (11.6)	17.1 (11.6)	23.7 (12.1)	25.4 (13.1)	26.4 (11.4)	8 × 10^−5^
**Total years smoked**; mean (SD)	44.3 (6.7)	42.1 (8.0)	44.2 (6.9)	45.5 (6.2)	45.3 (6.0)	44.7 (5.8)	0.03
**BMI**^e^, kg/m^2^; mean (SD)	27.6 (4.9)	29.2 (4.8)	27.6 (4.7)	27.6 (4.7)	27.7 (5.8)	26.3 (4.4)	0.004
**Race,** white; No. (%)	307 (97)	58 (98)	73 (94)	59 (100)	47 (96)	70 (99)	0.56^f^
**Sex**, female; No. (%)	109 (34)	21 (36)	25 (32)	26 (44)	15 (31)	22 (31)	0.60
**Intervention arm,** assigned to active; No. (%)	164 (52)	30 (51)	41 (53)	31 (53)	29 (59)	33 (46)	0.79
**Asbestos exposure**; No. (%)	51 (16)	15 (25)	14 (18)	7 (12)	5 (10)	10 (14)	0.05
**Stage;** No. (%)							0.66^g^
**Early stage (I/II)**	72 (23)	13 (18)	21 (29)	8 (11)	10 (14)	20 (28)	
**Late stage (III/IV)**	195 (62)	38 (19)	44 (23)	42 (22)	31 (16)	40 (21)	
**Unknown stage**	49 (16)	8 (16)	13 (27)	9 (18)	8 (16)	11 (22)	
**Histotype**; No. (%)							
**Adenocarcinoma**	132 (42)	28 (47)	35 (45)	20 (34)	18 (37)	31 (44)	
**Squamous cell carcinoma**	103 (33)	15 (25)	23 (29)	16 (27)	20 (41)	29 (41)	
**Small cell carcinoma**	71 (22)	15 (25)	19 (24)	19 (32)	9 (18)	9 (13)	
**Any death (through 2015)**; No. (%)	304 (96)	58 (98)	73 (94)	56 (95)	49 (100)	68 (96)	0.92^f^
**Years between blood draw and diagnosis**; mean (SD)	4.3 (2.5)	3.6 (2.5)	4.3 (2.5)	4.5 (2.4)	4.4 (2.6)	4.8 (2.3)	0.02
**Controls**							
	**(*n* = 316)**	**(*n*** **= 63)**	**(*n*** **= 64)**	**(*n* = 63)**	**(*n* = 63)**	**(*n* = 63)**	***P-*trend**^**b**^
**cg05575921**, % methylation; mean (SD)	69.3 (14.8)	90.8 (4.0)	78.3 (2.9)	67.6 (2.6)	60.0 (2.0)	49.4 (5.0)	–
**Age at blood draw**, years; mean (SD)	63.5 (5.7)	64.6 (6.0)	63.6 (5.2)	64.2 (5.8)	61.7 (5.9)	63.4 (5.4)	0.07
**Current smoker**; No. (%)	205 (65)	14 (22)	28 (44)	49 (78)	58 (92)	56 (89)	4 × 10^−25^
**Years since smoking cessation**^c^; mean (SD)	6.6 (6.4)	9.0 (7.9)	5.6 (4.3)	4.1 (3.4)	2.8 (2.6)	3.9 (4.5)	0.001
**Pack years**; mean (SD)	54.2 (24.1)	48.4 (23.5)	53.6 (18.8)	52.9 (23.2)	53.7 (25.8)	62.2 (27.2)	0.004
**Average cigarettes per day**^d^; mean (SD)	21.5 (10.7)	20.9 (8.6)	19.1 (10.9)	21.2 (9.7)	19.7 (9.9)	25.0 (11.9)	0.04
**Total years smoked**; mean (SD)	42.5 (7.4)	38.6 (8.0)	41.0 (6.9)	43.8 (6.9)	43.2 (7.3)	45.8 (6.0)	1 × 10^−8^
**BMI**^**e**^, kg/m^2^; mean (SD)	28.1 (5.5)	29.2 (5.0)	29.3 (6.9)	27.6 (5.4)	27.7 (5.4)	26.6 (4.3)	0.002
**Race,** white; No. (%)	307 (97)	62 (98)	59 (92)	61 (97)	62 (98)	63 (100)	0.19^f^
**Sex**, female; No. (%)	109 (34)	27 (43)	20 (31)	21 (33)	24 (38)	17 (27)	0.19
**Intervention arm,** assigned to active; No. (%)	168 (53)	27 (43)	32 (50)	36 (57)	38 (60)	35 (56)	0.07
**Asbestos exposure**; No. (%)	51 (16)	18 (29)	12 (19)	5 (8)	12 (19)	4 (6)	0.003
**Any death (through 2015)**; No. (%)	187 (59)	33 (52)	33 (52)	40 (63)	41 (65)	40 (63)	0.07

*Abbreviations*: *BMI* body mass index, *NSCLC* non-small cell lung cancer, *NSCLC, NOS* non-small cell lung cancer, not otherwise specified, *SD* standard deviation

^a^”Lung cancer cases” includes adenocarcinoma, squamous cell, and small cell, as well as 10 cases for whom histotype was NSCLC, NOS; other NSCLC; unknown or missing

^b^Linear trend tested using ordinal linear regression for continuous variables and Cochran-Armitage Trend Test for dichotomous variables across decreasing cg05575921 methylation quintiles

^c^Reported for individuals reporting former smoking status at blood draw (*n* = 111 case-control pairs)

^d^Reported for individuals reporting current smoking status at blood draw (*n* = 205 case-control pairs)

^e^BMI is missing for 1 case and 2 controls

^f^Fisher’s Exact test used due to at least 50% of cells containing expected counts of less than 5 per cell

^g^Linear trend by Cochran-Armitage Trend test for known stage only (early versus late; *n* = 293 cases)

Although strong and highly statistically significant associations were observed between decreased cg05575921 methylation and aspects of smoking exposure (Table 1; Additional file 1: Tables S1-S2), there were no clear associations between decreased cg05575921 methylation and lung cancer risk overall or by histotype in the 316 matched case-control pairs after controlling for age, estimated cell type, and cigarettes per day at blood draw (Table 2). Neither odds ratios nor linear trends reached statistical significance. While there was a non-statistically significant greater than two-fold increased risk of adenocarcinoma in Q2 and Q5 compared to Q1, there was no linear association (*P* = 0.50). All odds ratios for squamous cell carcinoma were below one, but they were statistically imprecise. Similar patterns were observed in the 242 case-control pairs where both members of the case-control pair would have been eligible for lung cancer screening per the 2014 USPSTF recommendations, with the exception of small cell histotype in which a borderline linear association emerged (*P-*trend = 0.05; Table 3). The screening-eligible small cell histotype quintile estimates became unstable due to small counts, but in the continuous model each 10% decrease in cg05575921 methylation was associated with a reduced small cell lung cancer risk (Odds Ratio (OR) = 0.51, 95% CI: 0.28–0.93). We did not observe interactions by sex.

**Table 2:** Lung cancer risk^a^ by cg05575921 percent methylation for all lung cancer cases and by histotype

	All lung cancer cases^**b**^			Adenocarcinoma			Squamous cell carcinoma			Small cell		
cg05575921 methylation %	Control	Case	OR (95% CI)	Control	Case	OR (95% CI)	Control	Case	OR (95% CI)	Control	Case	OR (95% CI)
	n	n		n	n		n	n		n	n	
**Continuous 10% decrease**	316	316	0.93 (0.79, 1.10)	132	132	1.10 (0.85, 1.42)	103	103	0.77 (0.54, 1.11)	71	71	0.70 (0.46, 1.07)
**Q1** (highest; hyper-methylated)	63	59	Ref	33	28	Ref	11	15	Ref	17	15	Ref
**Q2**	64	78	1.52 (0.81, 2.83)	23	35	2.41 (0.90, 6.46)	26	23	0.32 (0.07, 1.54)	13	19	2.40 (0.54, 10.60)
**Q3**	63	59	0.95 (0.47, 1.93)	27	20	1.48 (0.47, 4.63)	17	16	0.17 (0.03, 1.11)	16	19	1.08 (0.25, 4.60)
**Q4**	63	49	0.60 (0.28, 1.27)	32	18	0.95 (0.31, 2.89)	22	20	0.16 (0.02, 1.05)	8	9	0.30 (0.04, 2.42)
**Q5** (lowest; hypo-methylated)	63	71	1.03 (0.49, 2.16)	17	31	2.58 (0.77, 8.61)	27	29	0.23 (0.04, 1.37)	17	9	0.49 (0.07, 3.22)
	***P*-trend**		0.38	***P*-trend**		0.50	***P*-trend**		0.26	***P*-trend**		0.12

*Abbreviations*: *CI* confidence interval, *NSCLC* non-small cell lung cancer, *NSCLC, NOS* non-small cell lung cancer, not otherwise specified, *OR* Odds ratio

^a^Logistic regression model results, conditioned on matching factors (age at blood draw ±5 years, smoking status, sex, race, asbestos, enrollment year ±2 years, and time at risk) and adjusted for age at blood draw, estimated cell type, and cigarettes per day at blood draw

^b^“All lung cancer cases” includes adenocarcinoma, squamous cell, and small cell, as well as 10 cases for whom histotype was NSCLC, NOS; other NSCLC; unknown or missing

**Table 3:** Lung cancer risk^a^ by cg05575921 percent methylation, restricted to 2014 USPSTF^b^ lung cancer screening-eligible pairs

	All lung cancer cases^**c**^			Adenocarcinoma			Squamous cell carcinoma			Small cell		
cg05575921 methylation %	Control	Case	OR (95% CI)	Control	Case	OR (95% CI)	Control	Case	OR (95% CI)	Control	Case	OR (95% CI)
	n	n		n	n		n	n		n	n	
**Continuous 10% decrease**	242	242	0.90 (0.74, 1.08)	98	98	1.08 (0.79, 1.46)	82	82	0.76 (0.51, 1.12)	53	53	0.51 (0.28, 0.93)
**Q1** (highest; hyper-methylated)	40	43	Ref	21	21	Ref	7	11	Ref	11	10	Ref
**Q2**	51	59	1.05 (0.51, 2.16)	19	22	1.39 (0.42, 4.62)	21	21	0.27 (0.04, 1.88)	9	16	2.84 (0.32, 25.21)
**Q3**	51	44	0.77 (0.33, 1.79)	21	16	1.46 (0.35, 6.16)	14	12	0.12 (0.01, 1.42)	13	12	0.48 (0.05, 4.46)
**Q4**	46	40	0.55 (0.23, 1.34)	22	14	1.06 (0.28, 4.05)	17	17	0.13 (0.01, 1.41)	6	7	0.01 (0.00, 0.56)
**Q5** (lowest; hypo-methylated)	54	56	0.77 (0.33, 1.78)	15	25	1.87 (0.47, 7.46)	23	21	0.17 (0.02, 1.50)	14	8	0.52 (0.04, 6.22)
	***P*-trend**		0.29	***P*-trend**		0.55	***P*-trend**		0.23	***P*-trend**		0.05

*Abbreviations*: *CI* confidence interval, *NSCLC* non-small cell lung cancer, *NSCLC, NOS* non-small cell lung cancer, not otherwise specified, *OR* odds ratio, *USPSTF* United States Preventive Services Task Force

^a^Logistic regression model results, conditioned on matching factors (age at blood draw ±5 years, smoking status, sex, race, asbestos, enrollment year ±2 years, and time at risk) and adjusted for age at blood draw, estimated cell type, and cigarettes per day at blood draw

^b^Individuals aged 55–80 with at least 30 pack-year smoking histories who are current or former smokers who had quit within the past 15 years

^c^“All lung cancer cases” includes adenocarcinoma, squamous cell, and small cell, as well as 9 cases for whom histotype was NSCLC, NOS; other NSCLC; unknown or missing

In mortality analyses, decreasing cg05575921 methylation was borderline-statistically significantly associated with increased lung cancer-specific and all-cause mortality for all histotypes combined (*P*-trends = 0.05 and 0.06, respectively; Table 4). These associations were driven by the associations in adenocarcinoma and small cell histotypes; no association was observed for squamous cell carcinoma. Among adenocarcinoma cases, we observed linear associations between decreasing cg05575921 methylation quintiles and increased lung cancer-specific mortality (*P* = 0.01; Q5 vs Q1 HR = 2.32, 95% CI: 1.12–4.82) and all-cause mortality (*P* = 0.01; Q5 vs Q1 HR = 2.37, 95% CI: 1.20–4.71). Each continuous 10% decrease in cg05575921 methylation was associated with a 21% greater risk of death in adenocarcinoma cases (lung cancer-specific 95%CI: 1.03–1.43; all-cause 95% CI: 1.03–1.41). Among small cell cases, we observed a linear association between decreasing cg05575921 methylation quintiles and increased lung cancer-specific mortality (*P* = 0.04; Q5 vs Q1 HR = 3.68, 95% CI: 1.32–10.25), and although the all-cause mortality quintile results were generally similar, the linear trend was not statistically significant (*P* = 0.09). We did not observe evidence for statistical interaction by sex in any of our mortality models.

**Table 4:** Mortality^a^ by cg05575921 percent methylation for all lung cancer cases and by histotype

	All lung cancer cases^**b**^			Adenocarcinoma			Squamous cell carcinoma			Small cell		
cg05575921 methylation %	Deaths	Total	HR (95% CI)	Deaths	Total	HR (95% CI)	Deaths	Total	HR (95% CI)	Deaths	Total	HR (95% CI)
**Lung cancer-specific mortality**												
**Continuous 10% decrease**	313	372	1.08 (0.98, 1.19)	117	148	1.21 (1.03, 1.43)	94	115	0.98 (0.82, 1.17)	77	81	1.20 (0.93, 1.54)
**Q1** (highest; hyper-methylated)	59	74	Ref	23	31	Ref	15	19	Ref	16	19	Ref
**Q2**	61	74	0.93 (0.64, 1.36)	26	35	1.11 (0.58, 2.11)	20	23	1.02 (0.48, 2.14)	12	13	1.01 (0.37, 2.70)
**Q3**	63	75	1.12 (0.75, 1.67)	21	28	1.09 (0.54, 2.19)	12	17	0.89 (0.39, 2.03)	24	24	1.79 (0.79, 4.02)
**Q4**	65	74	1.13 (0.75, 1.71)	23	27	1.97 (0.95, 4.10)	23	27	0.86 (0.39, 1.92)	14	14	1.01 (0.35, 2.88)
**Q5** (lowest; hypo-methylated)	65	75	1.46 (0.95, 2.22)	24	27	2.32 (1.12, 4.82)	24	29	1.07 (0.49, 2.36)	11	11	3.68 (1.32, 10.25)
	***P*-trend**		0.05	***P*-trend**		0.01	***P*-trend**		1.00	***P*-trend**		0.04
**All-cause mortality**												
**Continuous 10% decrease**	357	372	1.07 (0.97, 1.17)	137	148	1.21 (1.03, 1.41)	113	115	0.97 (0.82, 1.14)	80	81	1.14 (0.89, 1.47)
**Q1** (highest; hyper-methylated)	73	74	Ref	30	31	Ref	19	19	Ref	19	19	Ref
**Q2**	69	74	0.87 (0.61, 1.24)	31	35	1.05 (0.58, 1.89)	23	23	1.10 (0.56, 2.20)	12	13	0.82 (0.31, 2.18)
**Q3**	71	75	1.07 (0.74, 1.56)	24	28	1.08 (0.56, 2.11)	17	17	1.11 (0.53, 2.31)	24	24	1.45 (0.66, 3.17)
**Q4**	70	74	1.01 (0.68, 1.49)	25	27	1.65 (0.83, 3.29)	26	27	0.78 (0.36, 1.66)	14	14	0.83 (0.30, 2.28)
**Q5** (lowest; hypo-methylated)	74	75	1.42 (0.96, 2.10)	27	27	2.37 (1.20, 4.71)	28	29	1.04 (0.50, 2.17)	11	11	2.95 (1.09, 7.96)
	***P*-trend**		0.06	***P*-trend**		0.01	***P*-trend**		0.76	***P*-trend**		0.09

*Abbreviations*: *CI* confidence Interval, *NSCLC* non-small cell lung cancer, *NSCLC, NOS* non-small cell lung cancer, not otherwise specified, *HR* hazard ratio

^a^Cox proportional hazards model results adjusted for age at blood draw, sex, years between blood draw and lung cancer diagnosis, and years since quit smoking at blood draw. All models include early, late, or unknown stage as a strata variable

^b^ “All lung cancer cases” includes adenocarcinoma, squamous cell carcinoma, and small cell cases as well as not otherwise specified non-small cell lung cancer (NSCLC, NOS; *n* = 16) and unknown/no pathology (*n* = 12)

Associations excluding individuals with 5PRIME extracted DNA were similar to the main risk and mortality results including them, respectively (Additional file 1: Tables S3-S5).

## Discussion

To our knowledge, our study is the first to examine associations between pre-diagnosis *AHRR* cg05575921 methylation and lung cancer risk and mortality by histotype among smokers at high risk of lung cancer. We observed that cg05575921 methylation differed dramatically by smoking exposure even among this population of heavy smokers, with mean pack years of 59.3 in cases and 54.2 in controls. Though strong and highly statistically significant associations were observed for lower cg05575921 methylation and greater smoking exposure in our study and in others [2–11], we did not observe that lower cg05575921 methylation was associated with an increased risk of lung cancer risk overall or by histotype. However, we observed that among lung cancer cases, decreased pre-diagnosis cg055759921 methylation was associated with increased mortality for adenocarcinoma and small cell, but not squamous cell lung cancer.

In prior epidemiologic publications, low levels of cg05575921 methylation have been associated with increased risks of lung cancer [4, 9, 19–22]. These reports include never and light smokers, and results have not been presented by histotype. In the population-based study by Bojesen et al. of approximately 23% never smokers and current/former smokers with mean smoking histories of fewer than 40 pack years, an over four-fold increased risk of lung cancer for individuals in the lowest versus highest methylation quintiles (95% CI: 2.31–10.30) was observed after adjusting for smoking status, cigarettes per day, and pack years [9]. In four publications reporting on combinations of study populations from up to five nested case-control studies, with each individual nested case-control study comprised of 63 to 367 pairs, statistically significant 40–60% increased risks of lung cancer per standard deviation decrease in cg05575921 methylation were reported [4, 19, 21, 22]. These results maintained statistical significance after adjustment for smoking for all but one study, which reported a statistically significant 63% increased risk that was attenuated and no longer statistically significant after controlling for smoking features (e.g., smoking status, pack years, comprehensive smoking index) [22]. In this study, cases had 20 mean pack years while controls averaged nine [22]. Our models of lung cancer risk in heavy smokers per standard deviation decrease in cg05575921 methylation were similar to the continuous 10% decrease model results shown in Table 2, with an OR = 0.91 (95% CI: 0.71–1.16) for the 316 case-control pairs after controlling for matching factors, age, estimated cell type, and cigarettes per day at blood draw.

In a study that performed a supplementary analysis restricting to the 2014 USPSTF screening eligible smokers, a non-statistically significant 1.2-fold increased risk of lung cancer per standard deviation decrease in cg05575921 methylation was observed after adjustment for age, sex, pack years, and time since quitting [20]. Again, there were large differences in smoking exposure by case control status, with mean pack years of 34 for cases and 13 for controls [20]. These results are in contrast to our results per standard deviation decrease in cg05575921 methylation, which were similar to the continuous 10% decrease model results shown in Table 3, with OR = 0.85 (95% CI: 0.65–1.13) in the 242 2014 USPSTF screening-eligible pairs after controlling for matching factors, age, estimated cell type, and cigarettes per day at blood draw. An update to the 2014 USPSTF screening guidelines is in process, with the 2020 draft USPSTF recommendation statement broadening eligibility by age (50–80 years) and smoking history (at least a 20 pack-year smoking history) [25]. Based on the 2020 draft USPSTF recommendation, 93% of the case-control pairs in our study would have been eligible for screening, and thus, our findings reflect the expected associations among that group.

Consistent with our observation that decreased pre-diagnosis cg05575921 methylation was associated with increased mortality in heavy smoker lung cancer cases, a case-cohort study with 60 fatal lung cancer cases in a subcohort of 1565 participants observed a multivariable-adjusted 1.56-fold increased hazard of lung cancer-specific death per 5% lower pre-diagnosis cg05575921 methylation (95% CI: 1.30–1.87) [23]. Histotype-specific results were not presented.

Decreased blood cg05575921 methylation is time- and dose-dependent on exposure to cigarette smoking, with cg05575921 methylation gradually increasing after a smoker quits smoking [11, 19, 36]. Two studies of former smokers have reported that cg05575921 methylation levels increase to never-smoker levels on average 10–22 years after cessation [19, 36], while two other studies report that decreased cg05575921 methylation persists 30–35 years post-cessation [11, 37]. Differences in length and condition of blood storage [38, 39], DNA extraction method [38, 40], and methylation quantification method [15, 41] may contribute to differences in cg05575921 methylation distributions across studies. Fortunately, such between-study differences do not tend to affect differential methylation detection across individuals on a per-study basis [15, 38–40]. This is supported by consistent replication of strong associations between low cg0557921 methylation with smoking features across studies [2–11], regardless of storage or processing.

A major strength of our study is that the population was at high risk of lung cancer due to high levels of cigarette smoke exposure. CARET selection was based on pack years smoked and time since cessation, and cases and controls were matched on current versus former smoking status at blood draw. While matching on smoking status may have ultimately limited our ability to see differences in risk and mortality with a marker that is so strongly related to smoking, our goal was to evaluate whether this marker provided information for lung cancer risk stratification above and beyond the effect of smoking.

## Conclusions

Although cg05575921 is a robust marker of cigarette smoking exposure, our results suggest that low levels of cg05575921 methylation are not associated with an increased risk of lung cancer in heavy smokers, and thus do not support using this marker for risk stratification for lung cancer screening among high-risk individuals. Additional research is needed to inform on whether decreased pre-diagnosis cg05575921 methylation is associated with mortality above and beyond smoking exposure, and thus may be useful for clinical decision making for lung adenocarcinoma and/or small cell lung carcinoma.

## Supplementary information

**Additional file 1: Table S1**. Characteristics of lung cancer cases (*n* = 372) by their quintiles of cg05575921 percent methylation. **Table S2**. Linear regression results for quintile cg05575921 hypomethylation and smoking features. **Table S3**. Lung cancer risk by cg05575921 percent methylation for all lung cancer cases and by histotype, excluding *n* = 3 case/control pairs where one had 5PRIME DNA extraction. **Table S4**. Lung cancer risk by cg05575921 percent methylation, restricted to 2014 USPSTF lung cancer screening-eligible pairs, excluding *n* = 1 case/control pair where one had 5PRIME DNA extraction. **Table S5**. Mortality by cg05575921 percent methylation for all lung cancer cases and by histotype, excluding *n* = 2 participants with 5PRIME DNA extraction.

**Additional file 2.** Participating CARET Institutions and Federalwide Assurance Numbers by Study Center.

## Data Availability

The data that support the findings of this study are available from CARET but restrictions apply to the availability of these data, which were used in agreement with CARET for the current study, and so are not publicly available. Data are available from the authors upon request and with permission of CARET (http://www.compass.fhcrc.org/caretWeb/requests/requesting.aspx).

## Abbreviations

*AHRR*: Aryl-hydrocarbon receptor repressor gene
BMI: Body mass index
CARET: β-Carotene and Retinol Efficacy Trial
CI: Confidence interval
CpG: Cytosine-guanine dinucleotide
EWAS: Epigenome-wide association studies
HR: Hazard ratio
NSCLC: Non-small cell lung cancer
NSCLC, NOS: Non-small cell lung cancer, not otherwise specified
PAH: Polycyclic aromatic hydrocarbon
OR: Odds ratio
SD: Standard deviation
USPSTF: United States Preventive Services Task Force
