# Supplementary material for: AHRR methylation in heavy smokers: associations with smoking, lung cancer risk, and lung cancer mortality
Source: BMC Cancer. 2020 Sep 22;20:905. doi: 10.1186/s12885-020-07407-x (PMC7510160; doi:10.1186/s12885-020-07407-x)
Supplement: Supplementary file 2 — Additional file 2. Participating CARET Institutions and Federalwide Assurance Numbers by Study Center. [file 12885_2020_7407_MOESM2_ESM.docx]

Additional File 2. Participating CARET Institutions and Federalwide Assurance Numbers by Study Center

| **Study center** | **Institution(s)** | **Federalwide Assurance Number (FWA)** |
| --- | --- | --- |
| Seattle | Fred Hutchinson Cancer Research Center, Seattle, Washington  University of Washington, Seattle, Washington | 00001920  00006878 |
| Baltimore | University of Maryland, Baltimore, Maryland | 00007145 |
| Portland | Kaiser Foundation Research Institute on behalf of Kaiser Foundation Hospitals  (Kaiser Permanente Center for Health Research, Portland, Oregon was the CARET-specific site) | 00002344 |
| New Haven | Yale University, New Haven, Connecticut  Lawrence & Memorial Hospital, New London, Connecticut | 00002571  00003097 |
| San Francisco | University of California, San Francisco, California | 00000068 |
| Irvine | University of California, Irvine, California | 00004071 |
